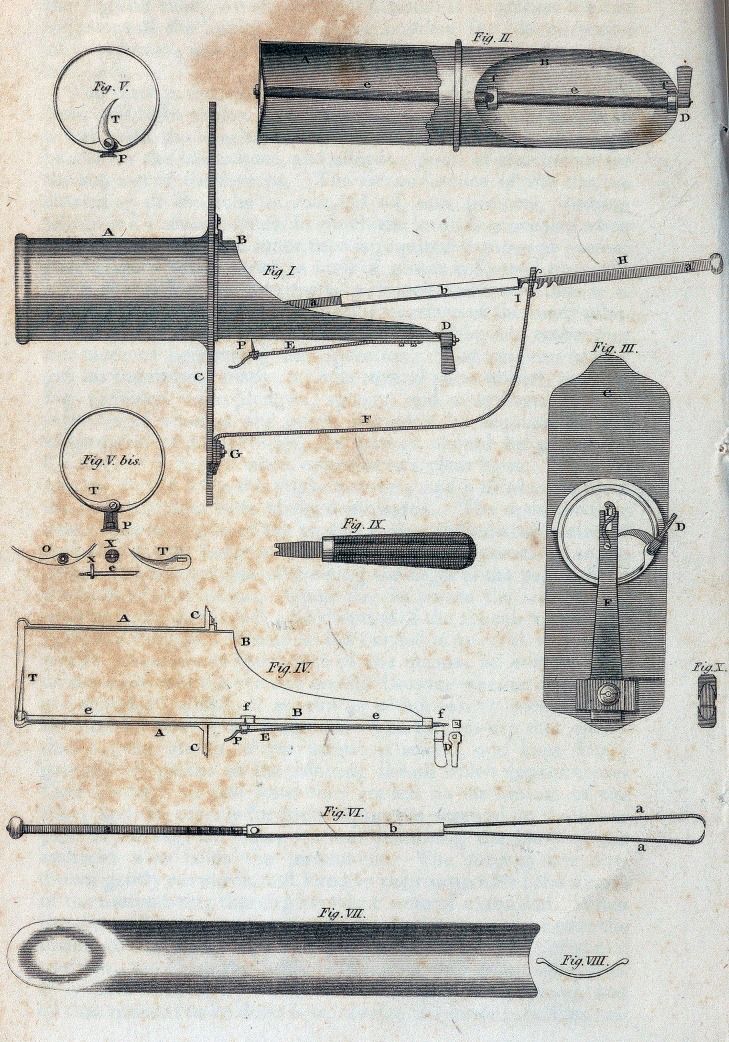# Description of an Instrument for the Extirpation of the Mouth and Neck of the Uterus, in Cases of Carcinomatous or Other Excrescences

**Published:** 1822-08

**Authors:** 

**Affiliations:** Riva di Trento.


### ORIGINAL COMMUNICATIONS,

### SELECT OBSERVATIONS, &c.

---

ART. I.—*Description of an Instrument for the Extirpation of the Mouth and Neck of the Uterus, in Cases of Carcinomatous or other Excrescences.* By Dr. CANELLA, of Riva di Trento.

DR. CANELLA was led to this contrivance in consequence of having a patient labouring under incipient carcinoma uteri, to which he found it necessary to apply caustic, by means of Recamier's speculum. Eight applications enabled him to destroy about one-third of the mouth of the womb; but the slowness of these operations left him no reasonable hope of a perfect cure, since, while his caustic acted upon the mouth of the affected viscus, the disease made proportionate advances towards its body. Being thus disappointed in his hopes, after having subjected his patient to considerable suffering, he resolved upon excision of the mouth and neck of the uterus. Before, however, the instrument devised for the purpose was brought to perfection, his patient was carried off by menorrhagia: his experiments were, in consequence, confined to the dead body.

As the speculum of Recamier is the foundation of this mechanism, and is not, we think, generally known in this country, it may be proper to prefix some account of it, with the particulars of the case for which it was first employed: the latter of which affords satisfactory proof of the practicability of destroying the neck of the womb by caustic, and the possibility of its amputation with safety to the patient.

A female, two years after her last confinement, laboured under fetid discharge from the vagina. On cohabiting with her husband, a small quantity of blood was voided; but without pain or interruption to conjugal enjoyment. On the commencement of December 1816, Mr. RECAMIER was consulted. Up to this period, the woman had experienced no pain. On examination *per vaginam*, a tumor was discovered, the size of an egg, with unequal surface: it was soft, pediculated, and situated on the anterior lip of the neck of the uterus, and was considered to be a cancerous fungus. Three days afterwards, Messrs. DUPUYTREN, DUBOIS, BOYER, and PELLETAN, were separately consulted, and entertained the same opinion respecting the carcinomatous nature of the tumor. From its rapid growth, the speedy destruction of the patient was a probable event; but, as it was circumscribed and confined to the anterior lip of the os uteri, and the remainder appeared to be sound, Messrs. Recamier and Dupuytren conceived that it might be extirpated with safety; and, that if a cure could not be effected, at least existence might be prolonged.

The operation was performed on the 15th of December, 1816, by M. Dupuytren. The patient being laid across the bed, two assistants supported and drew asunder the lower limbs; a third pressed upon the hypogastric region from above downwards. The mouth of the uterus was seized, and drawn to the vulva, by a pair of forceps; and the tumor, which consisted of a soft cerebriform mass, was removed by the curved scissars.

The incision was made upon a tissue which appeared sound, although a little more firm than the neck of the uterus is found to be in general. A small quantity of blood flowed after the operation, which was stopped by an injection of vinegar and water. No bad symptoms followed; and on the tenth day the patient was convalescent.

In April 1817, a cancerous excrescence, of the size of a nut, had formed on the posterior lip of the neck of the uterus, which was removed in the same manner; and in twelve days the patient resumed her usual occupations.

In May 1818, new vegetations were discovered, forming an unequal lobulated fungus. Mr. Recamier now conceived the idea of attacking it with caustic, and invented the instrument drawn in the margin, by which he could at once *see the diseased part*, and apply the remedy with complete safety to the surrounding vagina. This contrivance, which he denominates *speculum uteri*, is a simple metallic tube, and admirably fulfils the intention of its inventor. Its calibre should vary according to the size of the vagina. One extremity, which may be called uterine, is cut perpendicularly, and rounded at the margin to be applied to the neck of the uterus. The other extremity is sloped obliquely from above downwards.

At the end of May, Mr. R. made his first application of caustic. The affected parts having been brought into view by the tube, were touched by a camel's-hair pencil, dipped in a solution of nitrate of mercury; and the cauterized parts were covered with pledgits of lint, before it was withdrawn, to prevent the spreading of the caustic to the vagina. The pain produced by the application was moderate, and on the following day the patient felt no inconvenience from it. To promote the separation of the eschar, the mel. rosat. was applied in the same manner by the pencil. At the end of eight days, the sloughs separated, and the caustic was again had recourse to, which produced acute pain for the space of three hours. After fifteen cauterizations, thus repeated at intervals of about eight or ten days, the excrescences on the posterior surface of the lip were destroyed; but another yet remained, projecting nearly an inch from the anterior. The operator having now observed that, in consequence of this projection, a cul de sac was formed at the posterior part of the vagina, the rugæ of which were perceived, and were likely to be injected by the spreading of the caustic, the uterine extremity of the instrument was sloped off, by which it could be carried farther backward. Twelve cauterizations were required to destroy this. The neck of the uterus was entirely removed, and the caustic carried even to the body of the uterus. At the fourth month after this treatment was commenced, no further reproduction of the tumor had taken place, and hopes were entertained of a perfect cure; but the patient soon experienced a relapse. Lancinating pains were felt in the region of the uterus, which became intolerable, notwithstanding the strongest doses of opium, and she died in January 1820.*

Since the first employment of the instrument, Mr. Recamier has gone still further with it, having applied caustic, by its means, to a patient affected with three carcinomatous tumors in the rectum; the largest of which was ulcerated, and of the size of a nut.† It has undergone some modifications in the hands of Mr. Dupuytren, who has furnished it with handles, by which an assistant can retain it within the vagina, while the surgeon applies his remedies. Mr. Dubois has also varied it, by removing, through its whole length, that portion of the cylinder which corresponds to the upper part of the vagina, so as to enable him to detect and to treat urinary fistulæ.

Dr. Canella's instrument is, in fact, the speculum somewhat altered in form, with certain mechanism superadded, for the purpose of enabling the operator to draw down within it, and to keep in a state of moderate tension, the part to be removed; while, by means of a curved knife, (which, when not in action, is concealed at its uterine extremity,) the excision of the mouth of the womb can be effected by one turn of the hand. It is composed of two concentric tubes,—an external, or vaginal, and an internal, or exploratory; the latter being thus denominated, as the diseased parts are brought into view by it. The former should be of grain-tin, and proportioned in length and breadth to the dimension of the vagina. The latter may be of brass, or of some more firm metal, and should run accurately within the other, from the outer extremity of which it should

---

* *Dict. des Sciences Medicales,* vol. lii. p. 273.

† *Idem.* vol. xxxi. p. 243.

project four inches, and be sloped off, like the speculum; this increase of length being necessary, for the double purpose of affording a sight of the diseased part, and of enabling the operator to make a revolution of the tube in the operation. The lower part of this tube, at its inner surface, carries through its whole length a steel shaft, connected with it by two rings, so as to be capable of revolving freely. To the uterine extremity, both of tube and shaft, a curved knife is fixed, of sufficient length to admit of its point being plunged exactly into the centre of the diseased part, when the latter is brought within the tube, to the outer end of which a small handle is fixed, for the purpose of giving motion to the knife, by revolving the shaft. The knife, as we have before stated, is intended to be concealed when in a state of inaction: it should therefore correspond in shape with that part of the circumference of the instrument to which it is affixed.

When the handle hangs perpendicularly, the knife is in a state of concealment; by bringing the handle to a horizontal position, the knife takes an erect one, and is thus made to cut into the diseased substance which lies in its course; and it is there retained by the stopper of a spring placed at the under surface of the tube.

If the mouth and neck of the womb be brought exactly into the centre of the tube, and the knife be of suitable length, the point of the latter, when in action, will be passed into the centre of these parts. It will be obvious that, when the knife is here fixed, having already made a section of nearly one-fourth of their substance, a complete revolution of the inner tube upon its axis, made in a direction which will vary according to the situation of the cutting edge of the instrument, must effectually remove the remainder.

It is indispensable to the successful performance of the operation in question, that the part to be removed should be kept in a state of moderate extension, and fixed in the centre of the tube; since, without an attention to this particular, the knife would miss its aim in numerous instances; and in by far the greater proportion, if it should even pass into the part, it would not reach its centre, so that the revolution of the tube would not complete the excision. To obviate any inconvenience from this source, forceps are provided, whose blades are hooked at their extremities, in some respect after the manner of a double tenaculum; the mouth of the uterus is pierced by them at each side, and they are kept together by means of a running sheath. It is also desirable that the forceps, with the diseased part in their grasp, should be immovably fixed, without requiring the hand of the operator to hold them: this also is provided for by a branch, or supporter, which projects from a plate in front of the vaginal tube, whose extremity contains a groove for the lodgment of the forceps, which are notched for the purpose; and the branch is supplied with a kind of stop, to confine them within it.

The plate in front of the vaginal tube surrounds and goes off from it at right angles. Its longest diameter, when in use, is in a line with the longitudinal axis of the body: it serves as a handle for the instrument, and affords a point of attachment for the support of the forceps. The circumference of the uterine extremity of the tube is rounded off, and projects, forming within it a circular sulcus, in which the knife is concealed when not in action. As the inner tube is required to move in perfect parallelism with the outer, a kind of gutter is formed upon the plate surrounding the vaginal tube, which receives within it a corresponding prominence in the outer surface of the inner tube.

After this preliminary account, little need be said respecting the mode of performing the operation. The surgeon having got his instrument ready, with its several parts united, and being provided with pledgits, sponge, and a syringe, for the injection of vinegar and water, should it be required for the suppression of hemorrhage, the patient should be placed on the edge of a bed or table, opposite to a clear light. Two assistants should keep the thighs asunder, and a third press upon the hypogastrium from above downwards. The operator then passes into each side of the vagina a semi-cylindrical lamina of tin; this should bulge at the extremity which passes up to the uterus, for the purpose of drawing the rugæ of the vagina from the neck of this viscus. Upon this he passes the instrument, with its knife concealed, and divested of the branch which supports the forceps. When he has carried it up so as to contain within its extremity the mouth of the uterus, he entrusts it to an assistant, and the eye can readily discover whether any rugæ are included; which, if so, can be withdrawn by means of the tin lamina, now to be removed. The forceps are then introduced; the mouth of the uterus is seized, and kept firmly grasped by means of the running sheath which approximates their blades. In this state they are left on the bottom of the tube, and the branch for their support is screwed on the lower part of the vaginal plate, without disturbing the hand of the assistant who holds the instrument. The forceps are now drawn gently outwards, and fixed to their supporter; the mouth of the uterus being thereby kept in a state of extension. When the diseased parts are thus firmly fixed, the operator moves the handle of the shaft from the perpendicular to the horizontal position, by which the knife is plunged into their substance. It is there retained by means of the stopper of the spring, and all that remains to be done is to revolve the inner tube longitu-

Engraved for the London Med.l & Phys.l Journal & Published Aug.st 1. 1822. by J. Souter. 73. St Pauls Church Yard.

dinally upon its axis, which (according to the situation of the cutting edge of the knife, shown in the drawing,) must be effected in a direction downwards from the right side of the patient to the left. By this movement the part is completely cut off, and left within the branches of the forceps. The whole is now withdrawn from the vagina, which may be washed out by an injection of vinegar and water, and filled with pledgets of lint or sponge in case of hemorrhage.

EXPLANATION OF THE PLATE.

*Fig.* 1.—Metrotome, or instrument for the amputation of the womb with its several parts united.

A. Speculum, or vaginal tube.

B. Speculum, or internal exploratory tube, moveable.

C. Handles fixed to the vaginal tube, which serve also as the external boundary of the vaginal extremity of the tube; while the other extremity, furnished with a lip, may be called uterine.

D. A small steel handle, by which the shaft of the cutting part of the instrument is turned.

E. A spring, by which the point of P, the shaft belonging to the knife, is fixed when the latter is in action.

F. A branch from the handle of the vaginal tube, for the support of the forceps.

G. Screw for the union of the branch with the handle.

H. The forceps.

*aa.* Length of the forceps.

*b.* Sheath running upon the branches of the forceps.

*Fig.* 2.—Internal, or exploratory tube.

A. View of the inner portion of the tube, laid open.

B. Portion of the tube

D. Handle of the shaft, *ee.*

*ee.* The steel shaft seen through its whole extent.

*ff.* Rings united to the internal tube, to preserve the shaft in its situation, and to serve as a trochlea for it.

*Fig.* 3.—A front view of the instrument.

C. Handles fixed in the anterior, or vaginal extremity of the tube.

D. Handle of the shaft, by which the knife is put in motion.

I. A hook to secure the forceps in the notch of the branch F.

*Fig.* 4.—A lateral section of theinstrument.

AA. External, or vaginal tube.

BB. Internal, or exploratory tube.

CC. Handles of the vaginal tube.

D. Handle of the shaft.

*ee.* The shaft in its whole length.

*ff.* Rings of the shaft.

E. A spring which keeps the shaft in its situation, by means of the stopper P, when the knife is in action.

P. The stopper of the spring, which enters a hole in the shaft through an opening of the tube B.

T. Knife concealed.

*Fig*. 5.—Area of the inner tube.
T. Knife in action.
P. Stopper of the spring E introduced into the shaft.

*Fig*. 5, *bis*.
T. The knife concealed.
P. The stopper out of the shaft.
*o*. A lamina to be affixed to the circumference of the vaginal tube, for the concealment of the knife.
*e*. End of the shaft, to which the cutting blade is affixed.
*x, x*. Little nuts for the fastening of the cutting blade.

*Fig*. 6.—Forceps, resembling a double tenaculum.
*a*. Outward extremity, with notches by which they are lodged in the branch destined for its support.
*aa*. The branches of the forceps.
*b*. Running sheath, for the purpose of approximating the blades of the forceps.

*Fig*. 7.—Length and breadth of one of the conductors of the instrument.

*Fig*. 8.—Perspective view of the same, showing the thickness, size, and the concavity, of the uterine extremity.

*Fig*. 9.—Turnscrew, for the separation or re-union of the several parts of the instrument.

*Fig*. 10.—Perspective view of the extremities of the forceps.

## Figures and Tables

**Figure f1:**
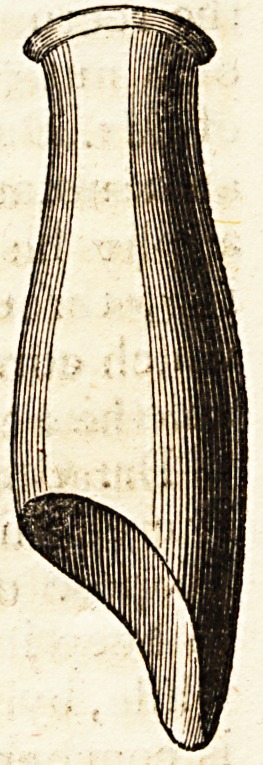


**Fig. V. Fig. II. Fig I Fig. V. bis. Fig. IX. Fig. III. Fig. IV. Fig. VI. Fig. VII. Fig. VIII. f2:**